# Collagen mutation and age contribute to differential craniofacial phenotypes in mouse models of osteogenesis imperfecta

**DOI:** 10.1093/jbmrpl/ziad004

**Published:** 2024-01-04

**Authors:** Hsiao H Sung, Wyatt J Spresser, Joseph P Hoffmann, Zongrui Dai, Peter M Van der Kraan, Michelle S Caird, Esmeralda Blaney Davidson, Kenneth M Kozloff

**Affiliations:** Orthopaedic Research Laboratories, Department of Orthopaedic Surgery, University of Michigan, Ann Arbor, MI 48109, United States; Department of Oral and Maxillofacial Surgery, University of Michigan, Ann Arbor, MI 48109, United States; Experimental Rheumatology, Department of Rheumatology, Radboud Medical Centre, Nijmegen, The Netherlands, 6525 GA; Department of Oral and Maxillofacial Surgery, University of Michigan, Ann Arbor, MI 48109, United States; Department of Oral and Maxillofacial Surgery, University of Michigan, Ann Arbor, MI 48109, United States; Department of Biostatistics, University of Michigan, Ann Arbor, MI 48109, United States; Experimental Rheumatology, Department of Rheumatology, Radboud Medical Centre, Nijmegen, The Netherlands, 6525 GA; Orthopaedic Research Laboratories, Department of Orthopaedic Surgery, University of Michigan, Ann Arbor, MI 48109, United States; Experimental Rheumatology, Department of Rheumatology, Radboud Medical Centre, Nijmegen, The Netherlands, 6525 GA; Orthopaedic Research Laboratories, Department of Orthopaedic Surgery, University of Michigan, Ann Arbor, MI 48109, United States

**Keywords:** osteogenesis imperfecta, maxilla, mandible, alveolar bone, teeth, micro-CT imaging

## Abstract

Craniofacial and dentoalveolar abnormalities are present in all types of osteogenesis imperfecta (OI). Mouse models of the disorder are critical to understand these abnormalities and underlying OI pathogenesis. Previous studies on severely affected OI mice report a broad spectrum of craniofacial phenotypes, exhibiting some similarities to the human disorder. The Brtl/+ and G610c/+ are moderately severe and mild-type IV OI, respectively. Little is known about the aging effects on the craniofacial bones of these models and their homology to human OI. This study aimed to analyze the Brtl/+ and G610c/+ craniofacial morphometries during aging to establish suitability for further OI craniofacial bone intervention studies. We performed morphological measurements on the micro-CT-scanned heads of 3-wk-old, 3-mo-old, and 6-mo-old female Brtl/+ and G610c/+ mice. We observed that Brtl/+ skulls are shorter in length than WT (*P* < .05), whereas G610c/+ skulls are similar in length to their WT counterparts. The Brtl/+ mice exhibit alveolar bone with a porotic-like appearance that is not observed in G610c/+. As they age, Brtl/+ mice show severe bone resorption in both the maxilla and mandible (*P* < .05). By contrast, G610c/+ mice experience mandibular resorption consistently across all ages, but maxillary resorption is only evident at 6 mo (*P* < .05). Western blot shows high osteoclastic activities in the Brtl/+ maxilla. Both models exhibit delayed pre-functional eruptions of the third molars (*P* < .05), which are similar to those observed in some bisphosphonate-treated OI subjects. Our study shows that the Brtl/+ and G610c/+ mice display clear features found in type IV OI patients; both show age-related changes in the craniofacial growth phenotype. Therefore, understanding the craniofacial features of these models and how they age will allow us to select the most accurate mouse model, mouse age, and bone structure for the specific craniofacial bone treatment of differing OI groups.

## Teaser

The Brtl/+ and G610c/+ are distinct osteogenesis imperfecta (OI) mouse models with unique collagen1 mutations. The Brtl/+ mice exhibit shorter skulls, porotic-like alveolar bone, and age-related bone resorption in both the maxilla and mandible. By contrast, G610c/+ mice not only resemble WT in alveolar bone appearance but also display age-related mandibular resorption. Both models show delayed third molar eruptions, resembling some OI patients. These model differences provide valuable insights for studying OI-related craniofacial bone treatments and the effects of aging on craniofacial features.

## Graphical Abstract

**Graphical Abstract:**
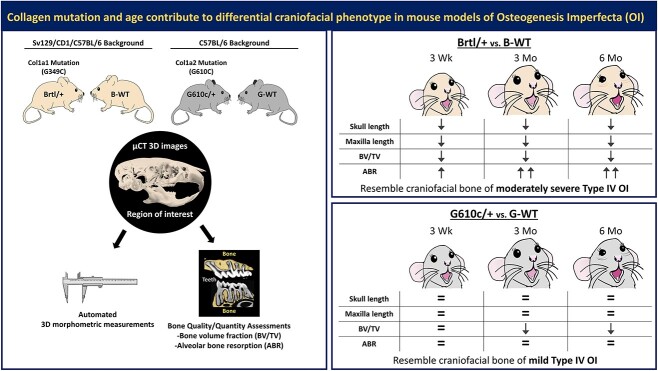

## Introduction

Osteogenesis imperfecta (OI) is a congenital disease characterized by bone dysplasia due to an abnormality in the synthesis and/or processing of the main protein of the bone extracellular matrix, type I collagen (Col1).^1^ The OI is mainly caused by *COL1A1* or *COL1A2* gene mutations^2^ and less commonly by mutations in gene encoding collagen-associated proteins (recessive forms).^3^ These mutations contribute to >22 OI types, with types I–IV being the most common.^4^ The Col1 is the most abundant protein found in bone.^5^ Defects in Col1 can result in bone quality, quantity, and/or morphologic alterations, as seen in OI.^5^ These Col1 alterations induce a unique facial appearance in OI, differing significantly from the non-OI population.

Type IV OI is highly variable, with symptoms ranging from mild to moderately severe bone deformity, reduced BMD, and abnormal bone microarchitecture.^6^^,^^7^ Fracture rates in type IV OI children tend to decrease with age, typically after puberty.^8^ Type IV OI subjects also exhibit abnormal craniofacial morphologies, such as relative macrocephaly, brachycephaly, variable dentinogenesis imperfecta expression, tooth agenesis, increased hypodontia, and poor dentoalveolar bone development.^7^^,^^9-14^

These high clinical heterogeneities are governed by the mutated gene, the mutation type, the mutation position along the gene, and the patient’s genetic background.^15^ Mouse models of the disorder are critical in understanding both the normal physiology, underlying OI pathogenesis, and response to medication therapies. The Brtl/+ and G610c/+ mice are type IV OI mouse models that are extensively studied in long bones for bone growth deficiencies, impaired fracture healing, defective mineralization of developing bone, and disrupted osteoblast differentiation.^16-20^ Both models have a glycine substitution in *Col1a1/2* genes, one of the most common mutation types in OI patients.^21^^,^^22^ The Brtl/+ mouse has *COL1A1* missense mutations that change an obligatory glycine to a cysteine at the 349th amino acid residue of the triple-helical domain.^23^ Similarly, the G610c/+ mouse has *COL1A2* missense mutations that change an obligatory glycine to a cysteine at the 610th amino acid residue of the triple-helical domain.^24^ Despite the similar mutation type, different causative genes, mutation positions, and mouse genetic backgrounds cause these 2 mice to exhibit different OI severities and aging characteristics. In our previous work examining Brtl/+ calvarial bones treated with the anabolic sclerostin antibody, we found that the genotype has a modest influence on skull shape.^25^ The Brtl/+ displayed a slightly larger, straighter lambda angle and a smaller, sharper bregma angle, resulting in a mildly domed skull appearance compared to the WT.^25^ However, there is a notable gap in the literature concerning craniofacial growth and development, especially of the maxilla and mandible, in both Brtl/+ and G610c/+ genotypes, with a particular emphasis on the effects of aging on these bones.

As humans, these mice have 2 distinct ossification modes, intramembranous and endochondral, that differentially develop the craniofacial and long-bone microarchitectures, respectively.^26^ Therefore, study results assessing long-bone morphology and aging characteristics in Brtl/+ and G610c/+ are not necessarily applicable to the craniofacial bone. Thus, this study aimed to analyze the Brtl/+ and G610c/+ craniofacial morphometrics and characteristics with aging to establish whether they are suitable models for studying efficient and safe OI craniofacial bone therapeutic interventions.

## Material and methods

### Experimental animals

The University of Michigan’s Committee on the Use and Care of Animals approved all protocols and procedures. All animals were bred and housed in an Association for Assessment and Accreditation of Laboratory Animal Care (AAALAC)-accredited vivarium. Female Brtl/+ mice have a mixed background of SV129/CD1/C57BL/6 and were derived from heterozygous Brtl/+ and WT parental strains. Female G610c/+ have a background of C57BL/6 and were derived from heterozygous G610c/+ and WT parental strains. Mice were euthanized at 3 wk (3w), 3 mo (3m), or 6 mo (6m) of age (Table 1).

**Table 1:** Study sample size per group.

**Sample size**				
Strain	**Female Brtl**		**Female G610c**	
Age/geno	**WT**	**Brtl/+(Het)**	**WT**	**G610c/+(Het)**
**3w**	11	10	11	10
**3m**	12	11	12	10
6m	10	10	10	10
**Total**	**33**	**31**	**33**	**30**

Sample size classified by strain, age and genotype. Abbreviations: 3w, 3-wk-old; 3m, 3-mo-old; 6m, 6-mo-old; Geno, genotype; Het, heterozygous.

### Micro-CT and analysis

A total of 127 mouse skulls were scanned with high-resolution micro-CT (μCT) (Bruker Skyscan 1176; Bruker) (Table 1). Image acquisition was performed using our previously reported protocol.^25^ Individual 2D cross-sectional images were reconstructed into 3D vol with 9-μm isotropic voxel size using NRecon software (version 1.6.5.8; Bruker). Dragonfly 2.0 (ORS) was used to delineate the ROI and to perform crania, maxilla, and mandible measurements, segmentations, and bone vol fraction analysis.

#### Craniofacial linear measurements

Measurements were performed on 3D reconstructed vol. Landmarks (Figure S1) were placed manually on the 3D reconstructed images, and the measurements were processed with an automated plug-in provided by ORS Dragonfly Team. Most of these parameters have been defined previously.^27^^,^^28^ All measurements were conducted by 3 users to ensure precision in landmark placement. The resulting standard errors across evaluators were <5%.

#### Maxilla alveolar bone vol fraction

Alveolar bone was evaluated at a ROI that includes anteroposteriorly the mesial side of the first molar to the distal side of the third molar, supero-inferiorly from the alveolar crest to the molar apex, and transversally from the buccal to palatal plates.

#### Alveolar bone resorption

The 3D reconstructions of the maxillary and mandibular teeth were standardized to the same scale value prior to resorption measurements using Image J (NIH). The bone recession was measured from the alveolar crest to the cementoenamel junction. Three measurements were conducted on each tooth (mesial, middle, and distal side of the tooth), totaling 9 measurements per sample.

#### Third molar eruption stage

The eruption stage of the third molar was determined by comparing the percentage emergence of its crown to the full eruption height. The full eruption height was measured from the alveolar crest (between the second and third molars to the occlusal plane of the second molar’s distal cusp). The third molar crown’s emergence was measured from the alveolar crest to its own occlusal plane. Crown emergence measurements for bilateral third molars were performed and then averaged. All measurements were performed using Image J software (NIH).

#### Interfrontal bone

We used 3D reconstructed μCT images to determine whether an ossicle was present within the interfrontal (metopic) suture for each sample. An ossicle was defined as an interfrontal bone (IFB) if completely separated from the frontal bone by the metopic suture. For samples with an IFB, we segmented both the frontal and IFBs, quantified their vol, and calculated the ratio of the IFB vol relative to the frontal bone vol.

### Western blot

Harvested maxillae underwent snap freeze in liquid nitrogen and stored at −80 °C until protein extraction. Maxillary alveolar bone protein extracts from 3w-, 3m-, and 6m-old Brtl+/− (*n* = 3 per age group) and WT littermates (*n* = 3 per age group) were obtained by grinding with Radioimmunoprecipitation assay (RIPA) lysis buffer. The protein concentration was measured using a BCA kit (Thermo Fisher Scientific). The sample solutions were heated at 95 °C for 5 min with 4× Nupage buffers. For each sample, proteins (20 μg) were separated on 4%–12% polyacrylamide gel and were then transferred onto a Polyvinylidene difluoride (PVDF) membrane (MilliporeSigma). Before immunodetection, gel sample loading was preliminary proved to be equivalent by Ponceau Red staining (0.2% w/v Ponceau S in 3% w/v trichloroacetic acid). The 5% non-fat dry milk and 0.05% Tween 20 were used to block the membrane, followed by overnight incubation with primary antibody: osteocalcin sc-376835 (Santa Cruz Biotechnology) as osteoblastic activity marker; Osteopontin sc-21742 (Santa Cruz Biotechnology) as late osteogenic differentiation marker; Nfatc1 sc-7294 Santa Cruz Biotechnology as osteoclastic differentiation marker; and Mmp9 af-909 (R&D Systems) as osteoclastic activity marker. Western blots were washed with 0.05% TBS-tween before 30 min room temperature incubation with anti-mouse secondary antibody sc-516102 (Santa Cruz Biotechnology San Jose). Immunostained bands were visualized using SuperSignal West Pico PLUS Chemiluminescent Substrate detection reagents (Thermo Scientific). Chemiluminescent signals were captured by ImageQuant LAS 4000 (GE Healthcare) digital imaging system. Band intensities were determined using an XRS+ Chemidoc system and Image Lab software (Biorad). The β-actin immunoblotting ensured an equal loading of samples.

### Statistical analysis

All height, width, and length data, comprising a total of 204 comparisons, including genotype, age, and mutant, were analyzed using nonparametric tests to avoid assumptions about variance or distribution. The Krustal–Wallis test was applied to determine whether there were any significant differences within multi-groups, and the post hoc Dunn test with Benjamin Hochberg correction was applied to determine the *P*-values for each comparison. These analyses were performed using R Statistical Software (v4.1.0, R Core Team 2020). All the other data were analyzed using 2-way ANOVA for the main effects of age and genotype. Differences between heterozygous and their corresponding WT were assessed using the student’s 2-tailed unpaired *t*-test, modified with Welch’s 2-sample *t*-test when interaction was present or to evaluate for type 1 error. These analyses and all graphs were performed using GraphPad Prism (v.9 for Windows, GraphPad Software). In all experiments, a *P*-value ≤ .05 was considered to be significant.

## Results

### Brtl/+ mouse has a shorter skull and facial length

**Figure 1:**
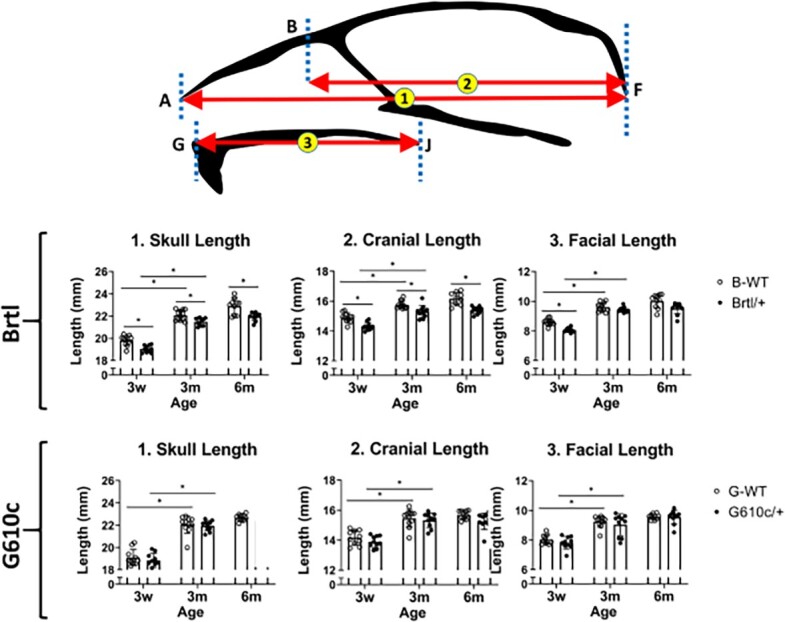
Craniofacial length measurements. Craniofacial length graphs, asterisk representing *P*-value ≤ .05. Points A = nasale, B = nasion, F = opisthion, G = Prosthion, and J = most caudal point of palatal bone. B-WT, Brtl WT, G-WT, G610c WT. Arrows illustrate the measurements.

**Figure 2:**
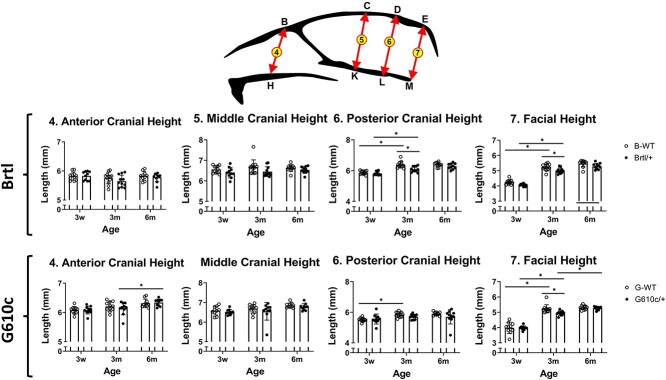
Craniofacial height measurements. Craniofacial height graphs, asterisk representing *P*-value ≤ .05. Points B = nasion, C=Bregma, D = intersection parietal bone with interparietal bone, E = intersection interparietal bone with squamous portion of the occipital bone, H = intersection maxilla and premaxilla, K = most ventral point of ISS, L = most ventral point of SOS, and M = most caudal point of basioccipital bone. B-WT, Brtl WT, G-WT, G610c WT. Arrows illustrate the measurements.

**Figure 3:**
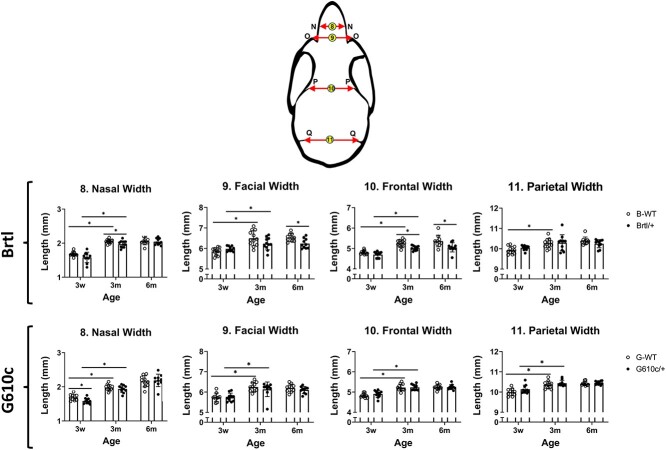
Craniofacial width measurements. Craniofacial width graphs, asterisk representing *P*-value ≤ .05. Points N = external margin of the nasal bone, O = anterior notch of the zygomatic process, P = frontal squamosal intersection, and Q = intersection of parietal-interparietal and occipital bones. B-WT, Brtl WT, G-WT, G610c WT. Arrows illustrate the measurements.

Both Brtl/+ and WT show skull anteroposterior growth with age. The average Brtl/+ skull lengths were 19.04 ± 0.31 mm, (3w), 21.48 ± 0.33 mm (3m), and 21.96 ± 0.36 mm (6m), which were significantly smaller than age-matched WT (19.80 ± 0.44 mm, 22.06 ± 0.46 mm, 22.90 ± 0.71 mm, respectively, *P* < .05) (Figure 1 and Figures S2 and S3). The average Brtl/+ cranial lengths were 14.31 ± 0.26 mm (3w), 15.30 ± 0.4 mm (3m), and 15.39 ± 0.24 mm (6m). These values were significantly smaller than age-matched WT (14.90 ± 0.32 mm, 15.76 ± 0.26 mm, 16.17 ± 0.40 mm, respectively, *P* < .05) (Figure 1 and Figures S2 and S3). Both, Brtl+ and WT exhibited significant progressive anteroposterior growth from 3w to 3m (skull length *P =* .006, cranial length *P =* .003). The skull and cranial length show a significant growth pattern between 3w and 3m. However, the growth rate decreases between 3m and 6m (Figure 1). No significant differences were found in the anterior and middle cranial heights between Brtl/+ and WT and across different age groups. However, Brtl/+ posterior cranial height is significantly smaller (6.10 ± 0.18 mm) than WT (6.38 ± 0.21 mm*, P* = .013) at 3m. This increase can be attributed to the significant growth in posterior cranial height observed in both groups at 3m (Figure 2 and Figure S2). The Brtl/+ has shorter facial length (8.06 ± 0.15) and height (4.07 ± 0.08) compared with WT (8.60 ± 0.22 and 4.25 ± 0.16, respectively, *P* < .05) at 3w. At 6m, the Brtl/+ facial measurements almost reach the WT measurements, except the facial width (6.24 ± 0.02.4), which is smaller than those of WT (6.53 ± 0.19, *P* = .04) (Figures 1–3 and Figure S3).

### G610c/+ mice display mild craniofacial disturbances

The average G610c/+ skull length (3w—18.84 ± 0.62. 3m—21.95 ± 0.46, and 6m—22.35 ± 0.64) is similar to WT (3w—19.10 ± 0.71, 3m—22.05 ± 0.47, and 6m—22.68 ± 0.29), and while slightly smaller, was not statistically significant (Figure 1 and Figures S2 and S3). Similar differences were observed in the G610c/+ cranial lengths (3w—13.90 ± 0.39. 3m—15.33 ± 0.49, and 6m—15.39 ± 0.24), similar to those found in WT (3w—14.17 ± 0.48, 3m—15.45 ± 0.58, and 6m—15.69 ± 0.28). No significant differences were found in the cranial heights between G610c/+ and WT. However, G610c/+ exhibited a significant increase in the anterior cranial height from 3m (6.14 ± 0.22) to 6m (6.34 ± 0.12, *P* = .042). On the other hand, the WT exhibited increases in posterior cranial height from 3w (5.86 ± 0.16) to 3m (5.90 ± 0.12, *P* = .013) (Figure 2 and Figure S3).

### Brtl/+ and G610c/+ exhibit a few craniofacial morphometric differences

Both the Brtl/+ and G610c/+ show similar skull and cranial lengths. However, there is a significant difference in cranial heights. The G610c/+ exhibits an increase in the cranial height (dome-shaped skull) across the age compared to Brtl/+. The G610c/+ tends to be more brachycephalic, primarily due to a wider frontal bone (3w—4.91 ± 0.17, 3m—5.23 ± 0.14, and 6m—5.25 ± 0.13) compared to Brtl/+ (3w—4.83 ± 0.10, 3m—5.22 ± 0.16, and 6m—5.23 ± 0.12, *P* < .05). The Brtl/+ tends to have narrower craniofacial widths, especially nasal width at 6m (2.04 ± 009) compared to G610c/+ (2.19 ± 0.18, *P* = .04). Additionally, Brtl/+ tends to have taller anterior mandibular heights than G610c/+, especially at 6m (1.85 ± 0.12, 1.66 ± 0.12, *P* = .016) (Figures S4 and S5).

### Brtl-WT tends to be larger than G610c-WT across all three age groups

There are no significant differences in skull length between Brtl-WT (B-WT) and G610c-WT (G-WT). However, the cranial length of B-WT (3w—14.90 ± 0.32 mm, 3m—15.76 ± 0.26 mm, and 6m—16.17 ± 0.40 mm) are longer than G-WT (3w—14.17 ± 0.48, 3m—15.45 ± 0.58, and 6m—15.69 ± 0.28), with significant differences observed at 3w (*P* = .007) and 6m (*P* = .03). In general, the craniofacial morphometrics, except for cranial height, tend to be larger in B-WT compared to G-WT (Figures S4 and S6).

### Brtl/+ mice have shorter, narrower, and pneumatized maxillary alveolar bone

**Figure 4:**
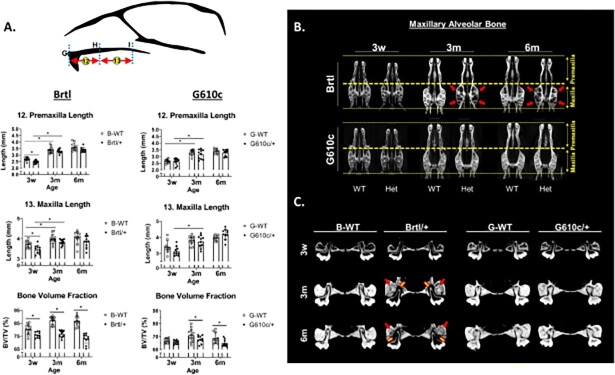
Maxilla measurements. (A) Premaxilla and maxilla lengths and alveolar bone vol fraction graphs, asterisk representing *P*-value ≤ .05. Points G = Prosthion, H = intersection maxilla and premaxilla, I = intersection maxilla and palatal bone. B-WT, Brtl WT, and G-WT, G610c WT. (B) Axial view showing the maxilla’s length and the alveolar bone’s appearance. The red arrow highlights the bone with a porotic-like appearance. (C) Coronal view showing the alveolar bone at the level of the first molar. The yellow arrow highlights the enlargement of the tooth sockets resulting from alveolar bone resorption, while the red arrow indicates the bone with a porotic-like appearance.

The premaxilla of Brtl/+ (3w—2.48 ± 0.12, 3m—3.30 ± 0.15, and 6m—3.39 ± 0.18) tends to be shorter than WT (3w—2.70 ± 0.13, 3m—3.39 ± 0.24, and 6m—3.62 ± 0.30) across all ages, with a significant difference observed at 3w (*P* = .013) (Figure 4A and B and Figure S3). The Brtl/+ maxilla (3w—3.46 ± 0.21, 3m—3.83 ± 0.14, and 6m—3.85 ± 0.29) also tend to be shorter than that of WT (3w—3.72 ± 0.17, 3m—3.99 ± 0.23, and 6m—4.05 ± 0.32), with a significant difference observed at 3w (*P* = .042) (Figure 4A and B and Figure S3). The G610c/+ premaxilla (3w—2.63 ± 0.24, 3m—3.16 ± 0.35, and 6m—3.29 ± 0.23) and maxilla lengths (3w—3.13 ± 0.29, 3m—3.73 ± 0.38, and 6m—3.97 ± 0.17) are similar to those of WT (3w—2.66 ± 0.14, 3m—3.30 ± 0.20, and 6m—3.39 ± 0.18; and 3w—3.41 ± 0.37, 3m—3.82 ± 0.27, and 6m—4.02 ± 0.32, respectively) (Figure 4A and B and Figure S3). The maxilla bone vol fraction (BV/TV) of the bones in the immediate vicinity of the maxillary teeth is reduced in the Brtl/+ (3w—75.99%, 3m—76.72%, and 6m—73.08%) compared with WT (3w—80.45%, 3m—87.29%, and 6m—86.32%, *P* < .05) (Figure 4A). The BV/TV of G610c/+ (65.63%) is similar to WT (66.80%) at 3w. With age, G610c/+ BVTV (3m—67.74% and 6m—64.80%) becomes smaller than WT (3m—70.87% and 6m—68.79%, *P* < .05) (Figure 4A). With age, Brtl/+ alveolar bone becomes more pneumatized (porotic-like appearance) (Figure 4B and C). The G610c/+ mice do not exhibit alveolar bone with a porotic-like appearance (Figure 4B and C).

**Figure 5:**
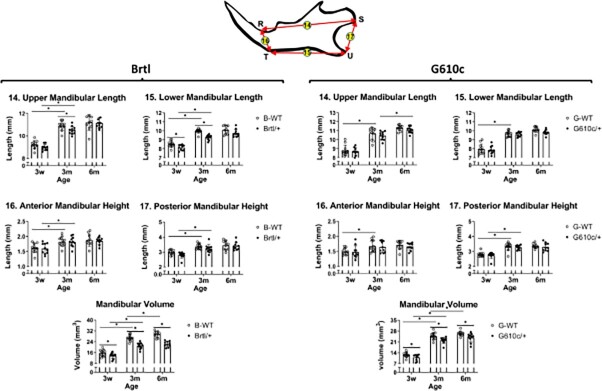
Mandibular measurements. Mandibular lengths, heights, and vol graphs. Asterisk representing *P*-value ≤ .05. R = superior rim of lower incisor alveolus (bone-tooth junction), S = most posterior point of the condyle, T = inferior rim of lower incisor alveolus (bone-tooth junction), U = tip of the mandibular angle. B-WT, Brtl WT, and G-WT, G610c WT.

### Brtl/+ and G610c/+ mandibles are slightly smaller compared to their corresponding WT

Brtl/+ tends to be slightly smaller compared to WT. This is mainly due to the significant anterior mandibular height reduction (3w—1.48 ± 0.21 vs 1.60 ± 0.18, 3m—1.64 ± 0.19 vs 1.83 ± 0.16, and 6m—1.66 ± 0.12 vs 1.85 ± 0.12 *P* = .017) at 6m (Figure 5 and Figure S3). The G610c/+ mandible also is overall slightly shorter compared to their WT, although it was not statistically significant (Figure 5 and Figure S3). The Brtl/+ mandibular vol (3w—13.72mm^3^, 3m—21.04mm^3^, and 6m—22.31mm^3^) is smaller compared with WT (3w—15.72mm^3^, 3m—26.88mm^3^, and 6m—29.54mm^3^, *P* < .05). The G610c/+ mandibular vol (3w—11.02mm^3^, 3m—22.06 mm^3^, and 6m—22.91mm^3^) is also smaller than their age-matched WT (3w—12.74mm^3^, 3m—24.82mm^3^, and 6m—26.45mm^3^, *P* < .05) (Figure 5). The Brtl/+ shows a smaller growth rate (3w–3m: 34.80%) than WT (3w–3m: 41.52%;). The G610c/+ exhibits a similar growth rate (3w–3m: 50.06%; 3m–6m: 3.71%) to WT (3w–3m: 48.69%; 3m–6m: 6.15%) (Figure 5). The G610c mutants are smaller than Brtl mutants at 3w (13.72mm^3^ vs 11.02mm^3^), but with aging, G610c/+ exhibit higher growth, surpassing the average size of the Brtl/+ (Figure S5). The WT-Brtl are larger than WT-G610c (3w—15.72mm^3^ vs 12.74mm^3^, 3m—26.88mm^3^ vs 24.82mm^3^, 6m—29.54mm^3^ vs 26.45mm^3^) (Figures S4 and S6). Overall, the biggest mandibular growth happens between 3w and 3m, which slows down between 3m and 6m. Like the maxilla, the mandibular alveolar bone exhibits a porotic-like appearance that is not observed in WT or G610c (Figure S7).

### Aged Brtl/+ and G610c/+ have severe alveolar bone resorption

**Figure 6:**
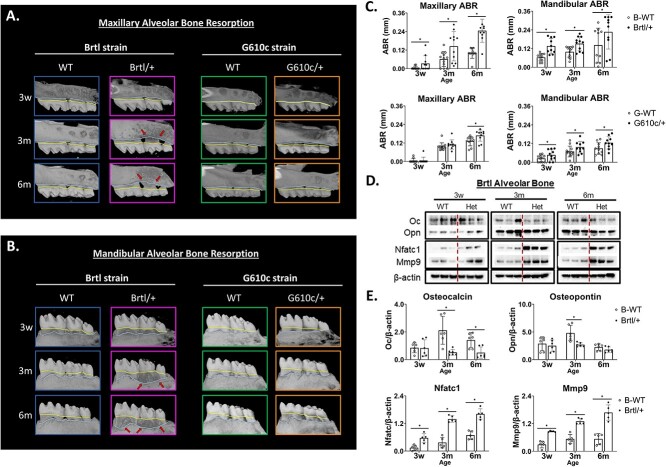
Alveolar bone resorption. (A) Sagittal μCT images of maxillary alveolar bone showing bone resorption by genotype and age, with cyan line depicting the alveolar bone level and yellow line depicting the cementoenamel junction; red arrows indicate severe resorption sites. (B) Sagittal μCT images of mandibular alveolar bone showing bone resorption by genotype and age, with cyan line depicting the alveolar bone level and yellow line depicting the cementoenamel junction; red arrows indicate severe resorption sites. (C) Brtl and G610c maxillary and mandibular alveolar bone resorption graphs, with asterisk representing *P*-value ≤ .05. (D) Western blot of Brtl alveolar bone. (E) Western blot graphs asterisk representing *P*-value ≤ .05.

The Brtl/+ maxilla (3w—0.01 mm, *P* = .01; 3m—0.07 mm, *P* ≤ .01; 6m—0.11 mm, *P* ≤ .01) and mandibular (3w—0.07 mm, *P* ≤ .01; 3m—0.10 mm, *P* ≤ .01; 6m—0.15 mm, *P* = .03) alveolar bones exhibit signs of resorption that worsen with age. This progressive resorption compromises more teeth with aging (Figure 6A–C). This resorption is corroborated with western blots that show decreased osteoblastic and increased osteoclastic activities in Brtl/+ alveolar bone that becomes exacerbated with age (Figure 6D and E). The G610c/+ mice show mild alveolar bone resorption with aging compared to Brtl/+. While this resorption becomes significantly more accentuated at 6m compared to earlier ages, resorption remains less severe than Brtl/+ (Figure 6A–C).

### Brtl/+ display delayed third molar eruption

**Figure 7:**
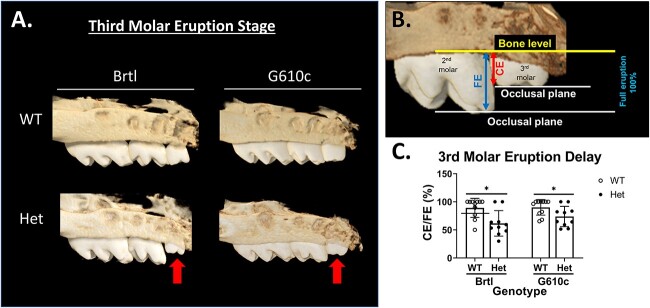
Third molar eruption delay. (A) 3D μCT reconstruction of 3w Brtl and G610c maxilla third molars (representative mean sample); the red arrow indicates the third molar crown emergence height. The blue arrow indicates the full eruption height measured from the alveolar crest to the occlusal plane of the second molar distal cusp. (B) Schematic that illustrates the method used to calculate the crown emergence (CE). (C) Graph illustrates the percentage of third molar CE of Brtl and G610c, with asterisk representing *P*-value ≤ .05.

Both Brtl/+ and G610c/+ mice show a significant pre-functional eruption delay of their third molars. From the analyzed samples, the average crown emergence for Brtl/+ was 68.8% compared to 90.4% of their littermate WT (*P* = .01). The average crown emergence of G610c/+ was 68.8%, contrasting with 95.2% of their WT (*P* = .03). Among the Brtl/+ third molars that have not reached the occlusal plane, the crown emergence was 68.8%. For G610c/+, it was 79.1% (Figure 7A–C).

### Brtl/+ and G610c/+ mice exhibit IFBs

**Figure 8:**
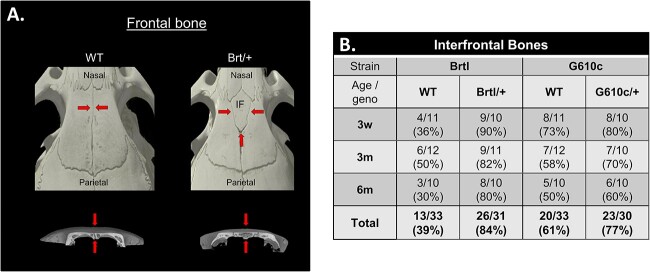
Interfrontal bone (IFB). (A) μCT images of 3D reconstructed WT and Brtl/+ skull showing the presence of IFB in between the frontal bone. (B) Summary table of the IFB in Brtl and G610c at each age. Geno, genotype.

During morphometric measurements, we observed the presence of a midline ossicle (Figure 8A). The IFB was observed in 84% of the Brtl/+ and 77% of the G610c/+ calvarial bones (Figure 8B). By contrast, only 39% of B-WT and 61% of G-WT mice exhibit the presence of these IFBs (Figure 8B). When present, Brtl/+ IFB demonstrated a larger vol compared with those present in WT. By contrast, both G610c/+ mutant and WT exhibited a smaller IFB, with IFB, on average, being 2% of the corresponding frontal bone vol. The Brtl/+ IFB is 4.9% of their frontal bone vol, while WT is substantially smaller (0.8%).

### G610c/+ mice and anterior cranial compartment misalignment

A subset of 3w G610c/+ mice (*n* = 3) presented with anterior compartment deformation with misalignment of the midline (Figure S8). This unexpected finding was observed during the μCT analysis.

## Discussion

Mutations in Col1 commonly induce a unique facial appearance, differing significantly from the non-OI population. A detailed cephalometric radiographic analysis of OI patients reveals deficiencies in the anterior cranial base and shorter maxillary, palatine, and mandibular lengths compared to age-matched controls.^11^^,^^14^ The Brtl/+ reflects a moderate-to-severe type IV OI exhibiting overall reduced cranial, maxilla, and mandibular morphometric indices compared with WT. Some Brtl/+ craniofacial morphometric reductions are more pronounced at 3w and tend to reach WT values at 6m age. This “catch-up” pattern is similar to what we previously observed in the long-bone phenotype.^16-19^ This pattern could be explained by possible matrix-level adaptations that improve whole bone strength without corresponding increases in structural geometry, especially after mice reach puberty, generally after 42d of age.^29^^,^^30^ Notably, these observations resemble findings observed in OI patients, where fracture rates tend to decrease after puberty.^8^ The Brtl/+ mice show a reduced skull and cranial anteroposterior (A–P) length similar to those previously reported in Col1a1^jrt^/+ and OIM/− mice.^31^^,^^32^ Both the Col1a1^jrt^/+ and OIM−/−mice have been described with a craniofacial resemblance to severe type OI patients.^31^^,^^32^ Similar results were observed here in Brtl/+, but not in G610c/+ mice, which exhibited a milder craniofacial phenotype, and their overall cranial morphometric indices are very similar to WT. Despite Brtl/+ and G610c/+ having the same type of missense mutation in the Col1, they exhibit phenotype heterogeneity, likely due, in part, to different mutation positions (Brtl/+ at Col1a1 G349C and G610c/+ at Col1a2 G610C) and differing genetic backgrounds (Brt/+ from a mixed of SV129/CD1/C57BL/6 and G610c/+ from C57BL/6). This could also explain the lack of similarity between Brtl/+ and G610c to the *Crtap^−^/^−^* mice, which have a mutation in collagen-associated genes and display brachycephalic skull shape that is characterized by nasofrontal suture and facial bone fusions.^33^ This fusion leads to mid-face retrusion,^33^ which is not observed in the mice of this study.

The most substantial and significant morphometric changes were observed within each genotype and strain between 3w and 3m. Surprisingly, no significant differences were observed between 3m and 6m. This occurrence could be attributed to the fact that mice reach their maturation stage between 3m and 6m. It is plausible to suggest that, after the craniofacial growth peak, which typically occurs around postnatal 60d,^34^ a gradual growth rate deceleration occurs, resulting in fewer discernible differences during this later period.

Overall, we found that the B-WT craniofacial bones are larger than G-WT, particularly at 3w and 3m. It may be explained that these mice have different growth peaks. Eventually, around 6m, G-WT is able to reach close to the B-WT size. It seems G-WT mice exhibit a delayed growth peak compared to B-WT mice, eventually reaching a size that closely resembles the B-WT as they age. On the other hand, while the heterozygous mice initially exhibit similar craniofacial sizes at a younger age, these differences become more pronounced with aging. These observed differences between the strains highlight that, despite both Col1 mutations, the WT mice are not strain interchangeable. To ensure an accurate comparison, it is crucial to compare each mutant to its corresponding WT littermates.^35^

We observed a significant impact of aging on both the Brtl/+ maxilla and mandible, resulting in severe alveolar bone loss. This severe alveolar resorption has been described as decreased alveolar bone in a previous Brtl/+ mandibular study.^36^ We also observed a similar pattern of bone loss in 6m G610c/+ mice, while younger mice did not exhibit such changes. Similar reduced alveolar bone has also been reported in *crtap−/−* mice, which harbor mutations in collagen-associated genes.^33^ Furthermore, this alveolar bone loss is associated with an upregulation of osteoclastic activity, specifically Mmp9, and Nfatc1. These severe alveolar resorptions observed with aging may be secondary to the poor bone quantity and quality inherent to OI.^1^^,^^3^^,^^6^^,^^37^ Previous study shows that the Brtl/+ mice are more prone to microdamage accumulation compared to age-matched WT. This was evident when the B rtl/+ ulnae exposed to typical cage activities showed a significantly greater amount of cortical microdamage than those of the WT.^38^ If this observation extends to the maxilla and mandible bones, it can be assumed that high biomechanical loading from chewing cycles (occlusion) may exacerbate pre-existing microdamage in Brtl/+. Given this, it seems plausible to suggest that the increased alveolar resorption in Brtl/+ might result from this cumulative loading effect on the compromised Brtl/+ bone quality, which aligns with observations described in Col1a1^jrt^/+ mice.^31^

Interestingly, the maxillary and mandibular alveolar BV/TV mice exhibit a noteworthy reduction as both Brtl and G610c mice age, especially when comparing across the ages. This finding aligns with the reported observations in humans, where a decline in trabecular BV/TV has been consistently associated with the natural aging process.^39^

We found a delay in the upper third molar eruptions in both Brtl/+ and G610c/+ mice. Tooth eruption has two stages—intraosseous and supraosseous. The intraosseous stage requires osteoclastic activity to create the eruption path by removing the bone. Once the tooth cusps reach the alveolar crest, the eruption pathway is complete. Then, the tooth transitions to the supra osseous stage, which requires bone apposition apical to the developing tooth in order to push the tooth to the occlusal plane.^40^^,^^41^ Delayed tooth eruption has been found following the administration of bisphosphonates to newborn rats. Evidence for similar effects in humans is sparse, since only a few studies show tooth maturity and eruption delay in bisphosphonate-treated OI patients compared to non-OI children.^42^^,^^43^ As these studies compare the tooth eruption of OI patients with non-OI children instead of non-bisphosphonate-treated OI patients, bisphosphonates may not be the only factor affecting eruption in this population. Rather, the poor OI bone quantity and quality could also be participating in the tooth eruption delay. Poor OI bone may not produce proper and sufficient apical bone to induce the propulsion movement for tooth eruption at the same pace as non-OI subjects.

Our OI mice differed from *Crtap−/−* mice regarding cranial bone fusions. While cranial fusions disturbances were not observed in our mice, they did exhibit IFB ossicles, which was not observed in the *Crtap^−/−^* mice.^33^ As a radiographic finding, we observed a higher IFB in Brtl/+ and G610c/+ than in their corresponding WT counterparts. The IFB are small irregular bones that are present within sutures or fontanelles. Our IFB seems to be a Wormian bone. Wormian bones have been described as an idiopathic minor skeletal variant that could be present in numerous recognized syndromes.^44^ In fact, many OI subjects have an abnormally large number of Wormian bones visible on a skull radiograph.^45^ It has been proposed that abnormal mechanical stresses across cranial sutures are the common denominator for various conditions associated with the presence of Wormian bones.^45^ A previous study found the presence of Wormian bones in most OI types III and IV, but this was less in OI type I individuals.^45^ Wormian bones appear more frequently in more severely affected OI patients and seem to develop mostly in utero.^45^ Our Brtl/+ and G610c mice emulate type IV OI, and their interfrontal or Wormian bones are completely surrounded by the metopic suture, separating them from the frontal bones. However, previous studies on various inbred mouse strains observed the presence of an ossicle residing between the frontal bones.^46^ The IFB was described as a quasi-continuous trait that exhibits, when present, morphological variability. The C57BL6 and CBA mice also consistently exhibit IFB.^46^ The Brtl/+ has a mixed background, unlike the G610c/+, which is genetically more homogeneous. However, the presence of a C57BL6 background in both Brtl/+ and G610c/+ mice may explain the presence of the IFB due to genetic components in C57BL6 mice. Heterozygous mutants, however, show a higher incidence of IFB than WT counterparts, which are larger in size when measured related to their corresponding frontal bone sizes. Therefore, the presence of IFB could also be influenced by the Col1 mutation. A better understanding of the genetic and environmental factors that influence IFB development may be relevant to understanding the presence of OI Wormian bone formation.

In summary, our study shows that the Brtl/+ and G610c/+ mice display clear features found in type IV OI patients; more importantly, they show similar age-related changes in craniofacial growth phenotype. Based on our findings, these mild and moderately severe models can be used to study the mechanisms underlying the craniofacial abnormalities of OI patients. Therefore, understanding the craniofacial features of these models and how they age will allow us to select the most accurate mouse model, mouse age, and bone structure for the treatment of a specific craniofacial site of differing OI groups.

## Supplementary Material

Sung_final_manuscript_supplemental_publishing_edits_v1_ziad004

Figure_S2_ziad004

Figure_S3_ziad004

Figure_S4_ziad004

Figure_S5_ziad004

Figure_S6_ziad004

Figure_S7_ziad004

Figure_S8_ziad004

## Data Availability

The data that support the findings of this study are available from the corresponding author upon request.
