# Supplementary material for: Collagen mutation and age contribute to differential craniofacial phenotypes in mouse models of osteogenesis imperfecta
Source: JBMR Plus. 2024 Jan 4;8(1):ziad004. doi: 10.1093/jbmrpl/ziad004 (PMC11059998; doi:10.1093/jbmrpl/ziad004)
Supplement: Sung_final_manuscript_supplemental_publishing_edits_v1_ziad004 [file sung_final_manuscript_supplemental_publishing_edits_v1_ziad004.docx]

**
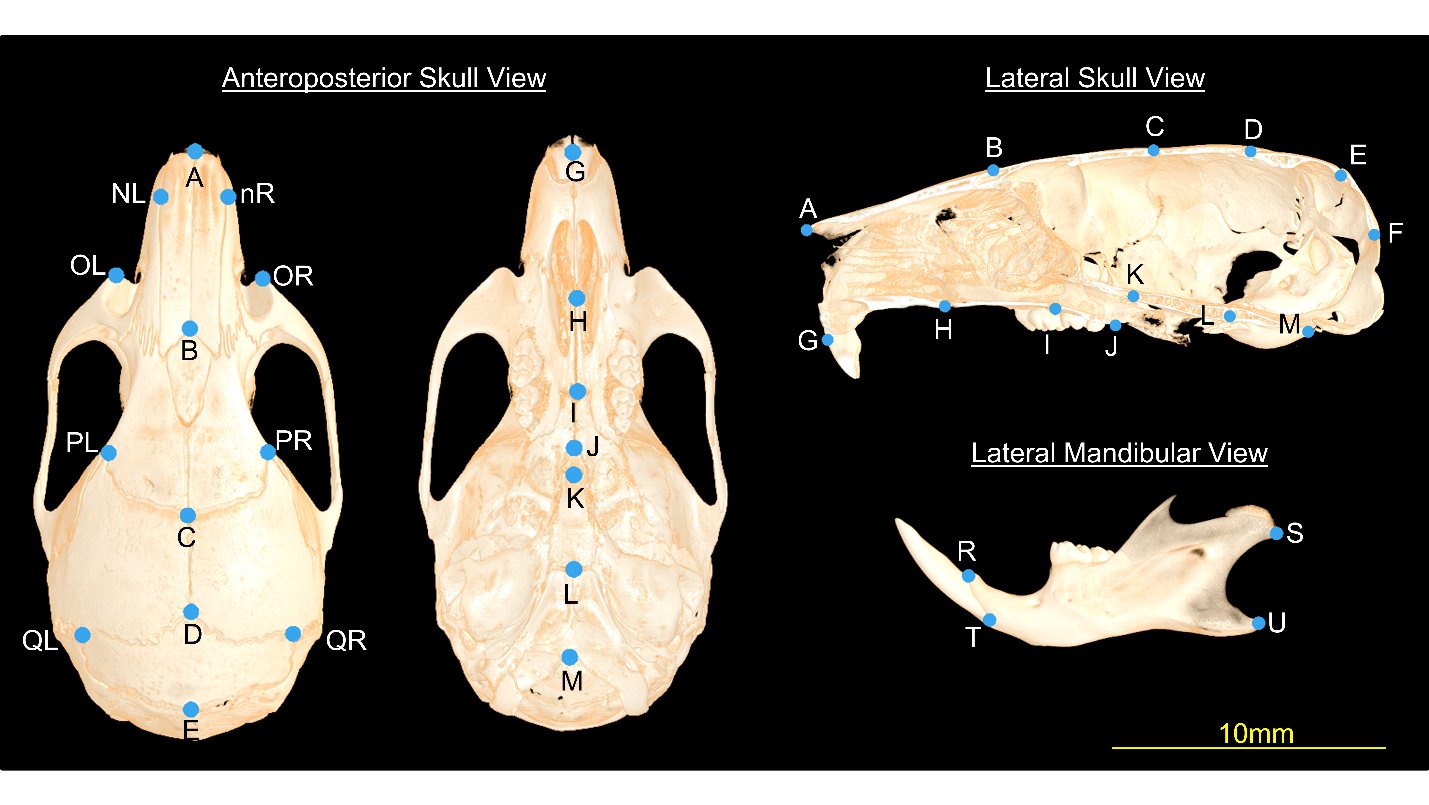
Supplemental Figure 1. Micro-CT Craniofacial landmarks**. The representative 3D craniofacial reconstruction images illustrate the landmark points utilized for automated craniofacial lengths, heights, and widths measurements.


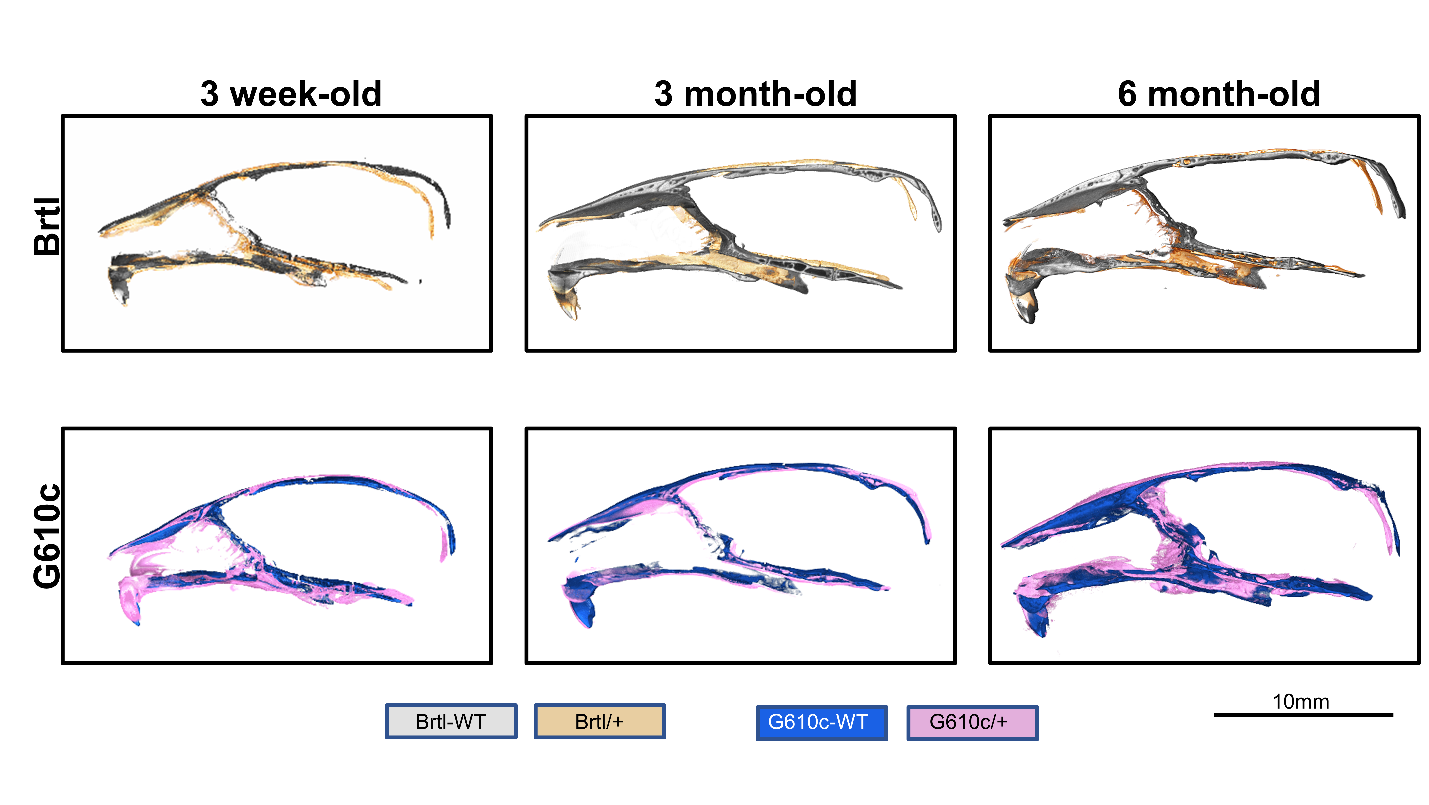


**Supplemental Figure 2. 3D Skull µCT reconstruction images overlapped by genotype**. The lateral view of the 3D µCT reconstruction images were obtained from the midsagittal slab, demonstrating the overlapping of the WT and Heterozygous (Het) samples from each age and mutation strain. The skulls overlapping were aligned based on the nasospinale and prosthion reference points. It shows the overlap of the WT and Het of each age and mutation strain. Representative 3D slabs were selected from samples that had the average length within each group. The silver line illustrates Brtl-WT, the orange line represents Brtl/+, the blue line illustrates G610c-WT, and the pink line illustrates G610c/+.

**
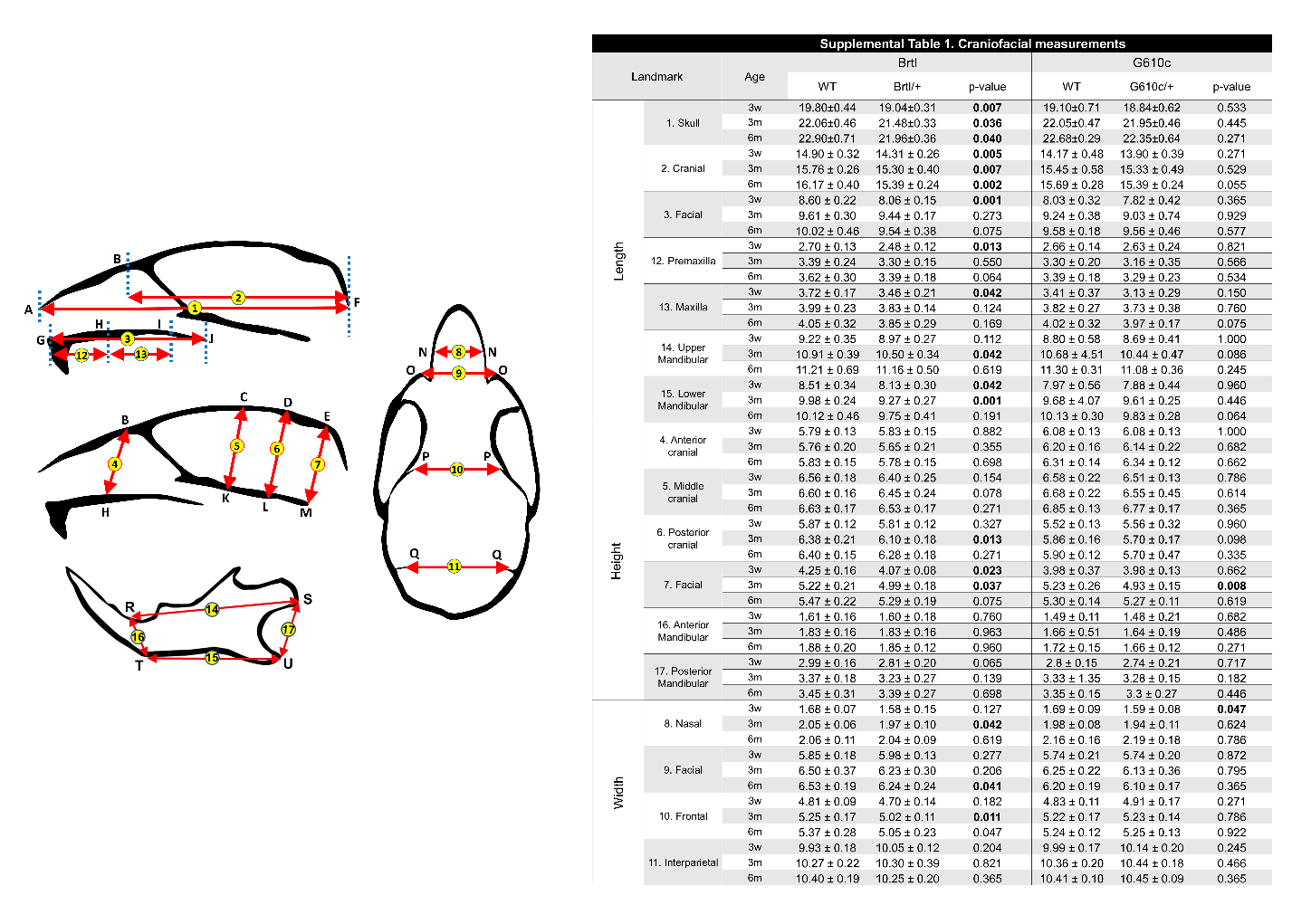
**

**Supplemental Figure 3. Genotype and age measurement results. A.** Figure showing the measurements analyzed in this study. **B.** Summary table showing genotype data (WT vs Het) categorized by age and mutation.

**
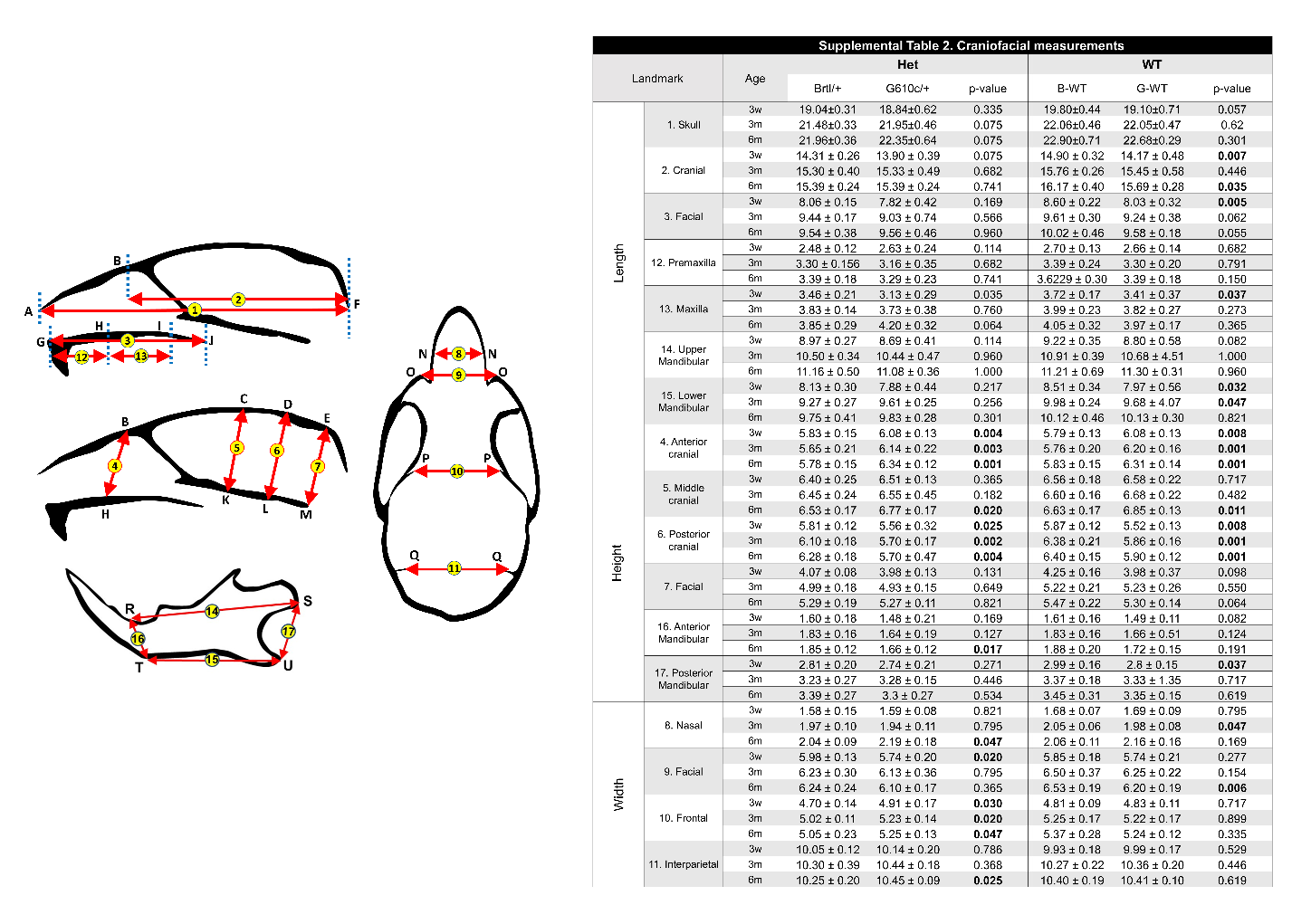
**

**Supplemental Figure 4. Mutation and strain measurement results. A.** Figure showing the measurements analyzed in this study. **B.** Summary table showing mutation data (Brt/+ vs G610c/+) and strain data (Brtl-WT vs G610c-WT) categorized by age.


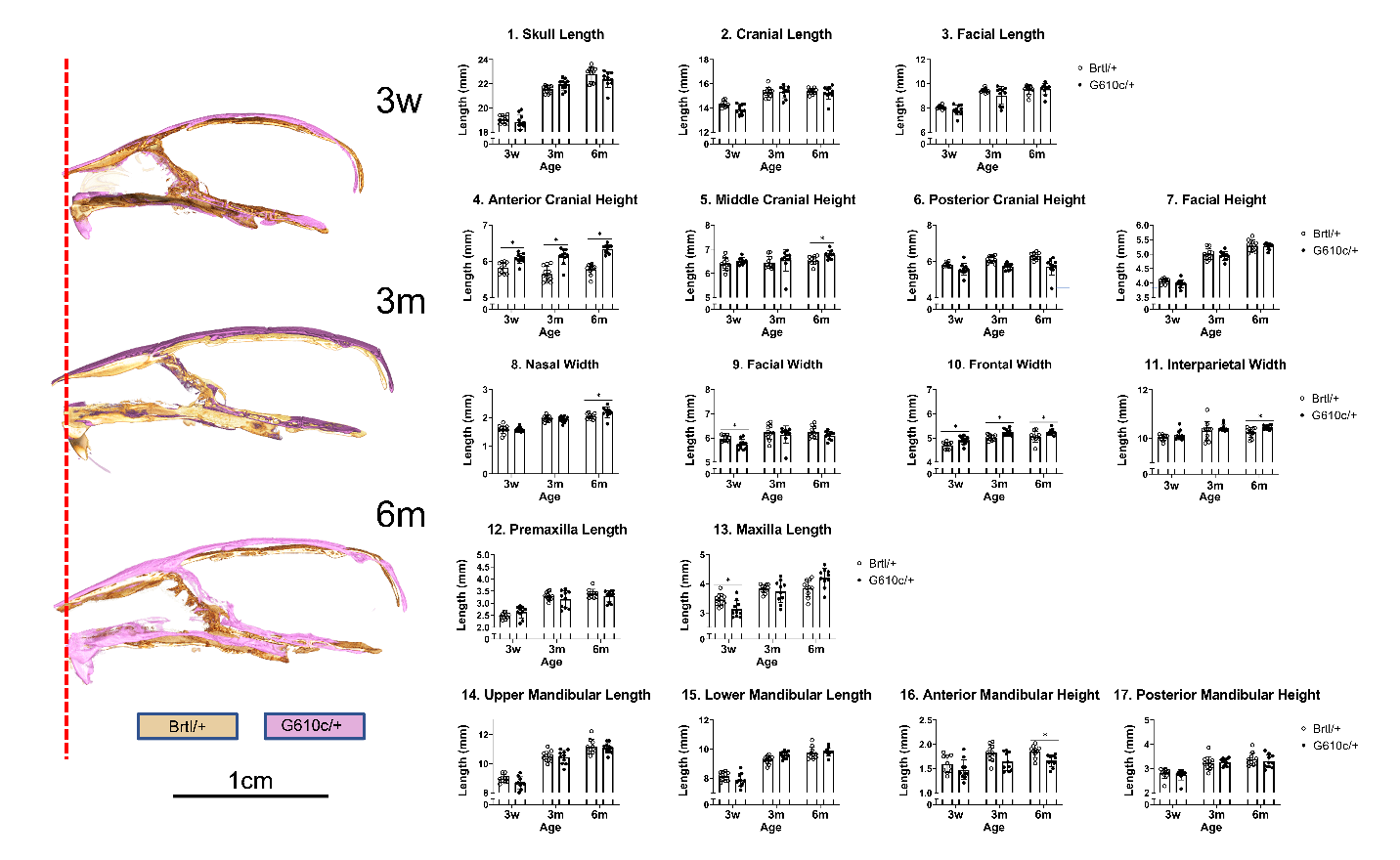


**Supplemental Figure 5. Brtl/+ vs G610c/+ Craniofacial measurements. A**. Representative 3D µCT images of Brtl/+ and G610c/+. Images are overlapped by age. **B.** Craniofacial measurement graphs, asterisk representing *p-value*≤0.05.

**
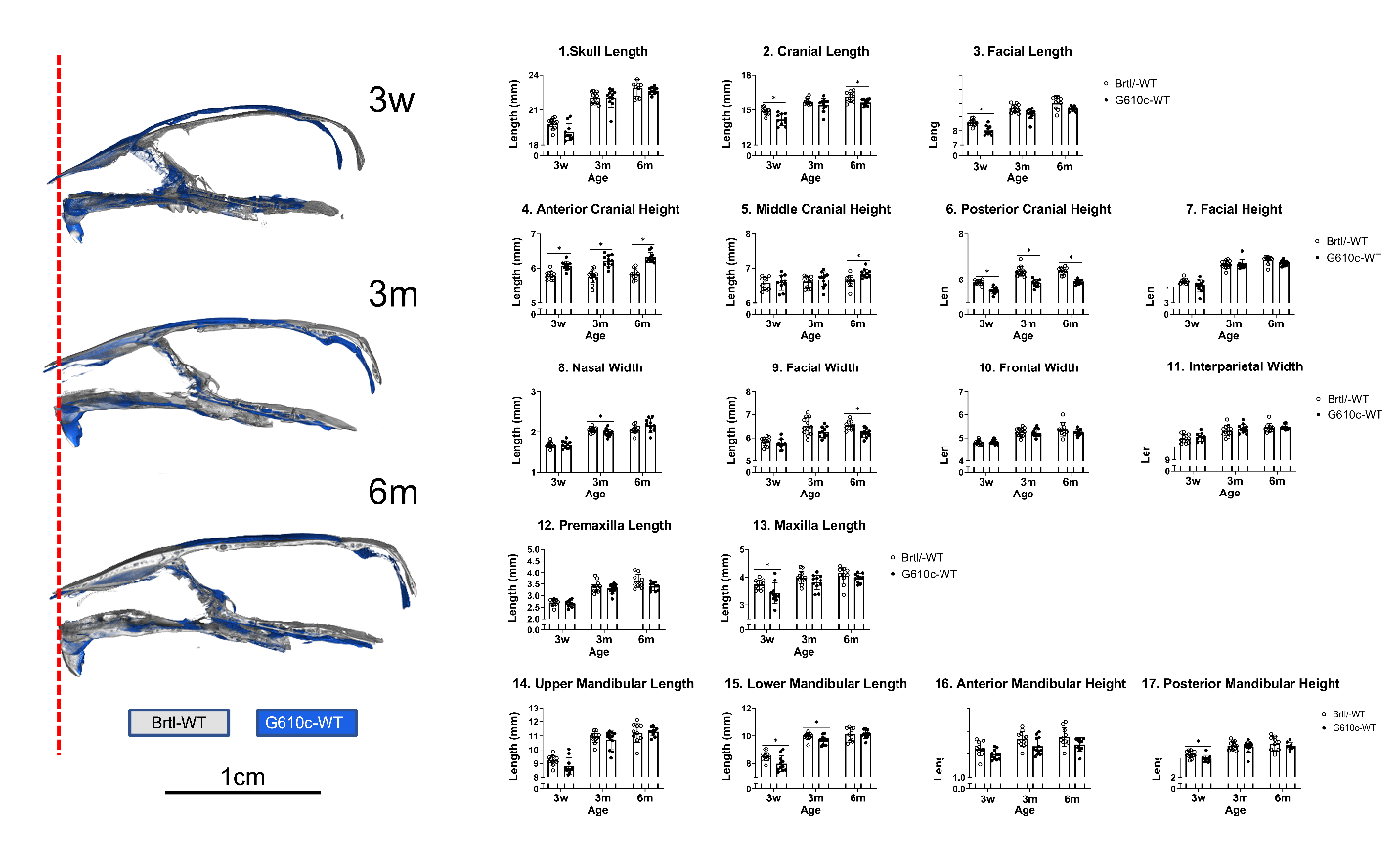
**

**Supplemental Figure 6. Brtl-WT vs G610c-WT Craniofacial measurements. A**. Representative 3D µCT images of Brtl-WT and G610c-WT. Images are overlapped by age. **B.** Craniofacial measurement graphs, asterisk representing *p-value*≤0.05.

**
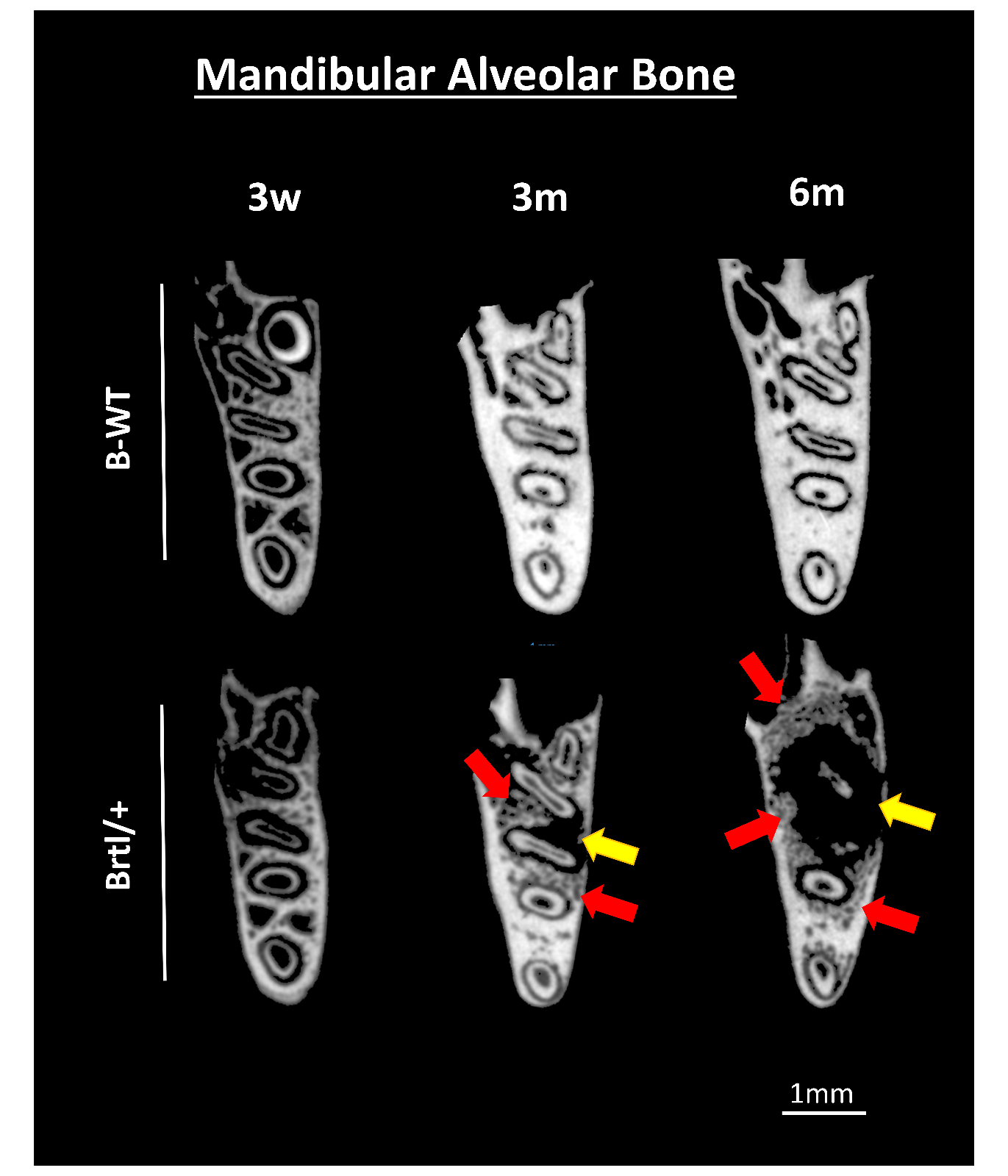
**

**Supplemental Figure 7. Mandibular alveolar bone resorption.** Axial view of the mandibular alveolar bone. The red arrow highlights the bone with a porotic-like appearance, and the yellow arrow highlights the enlargement of the tooth sockets resulting from alveolar bone resorption.


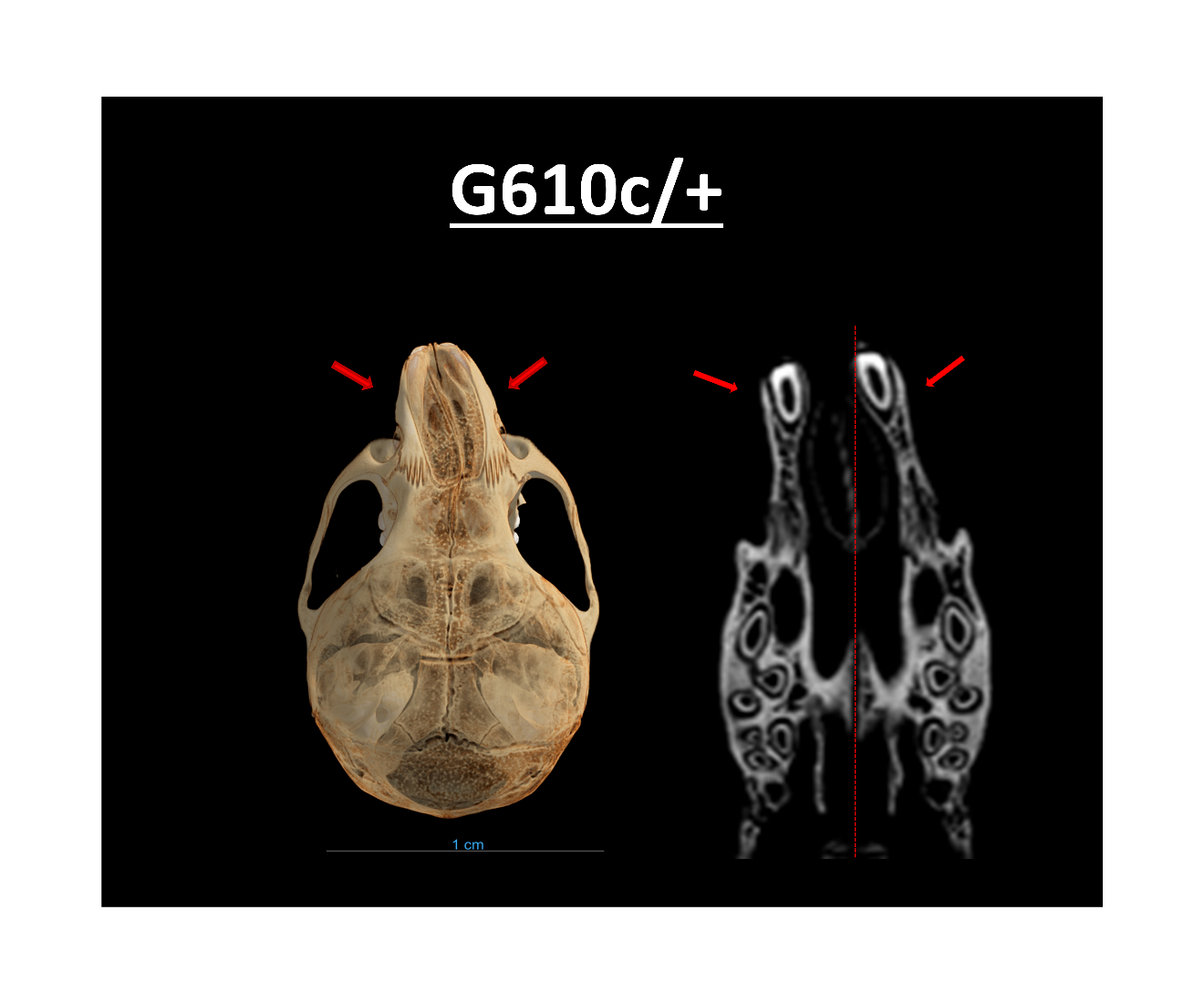


**Supplemental Figure 8. G610c/+ skull malformations.** Red arrows indicate the midline deviation in 3D µCT reconstruction and axial view of anterior cranial compartment malformations.
