# Supplementary figures and images for: Collagen mutation and age contribute to differential craniofacial phenotypes in mouse models of osteogenesis imperfecta
Source: JBMR Plus. 2024 Jan 4;8(1):ziad004. doi: 10.1093/jbmrpl/ziad004 (PMC11059998; doi:10.1093/jbmrpl/ziad004)

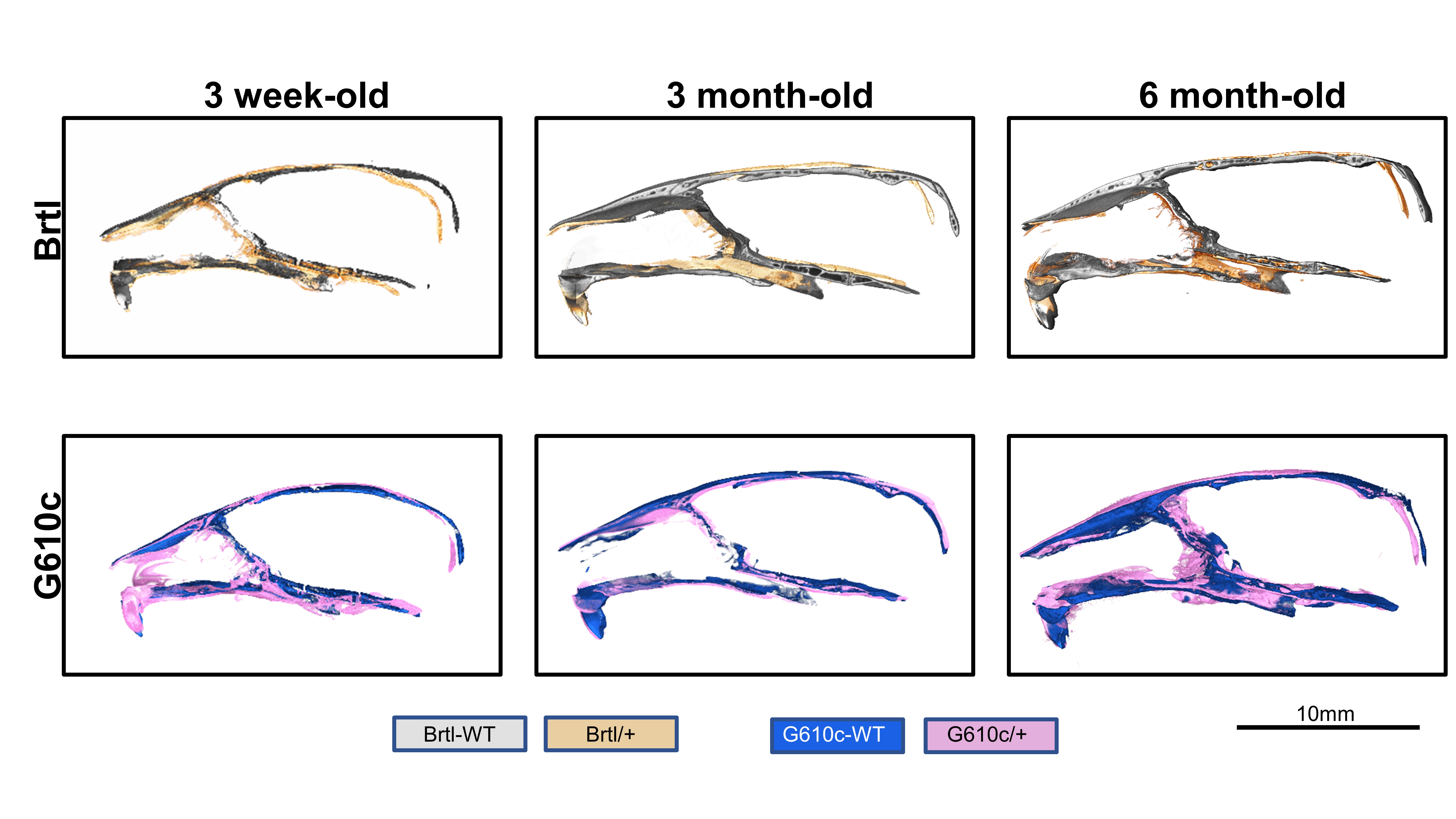

Supplement: Figure_S2_ziad004 [file figure_s2_ziad004.jpeg]

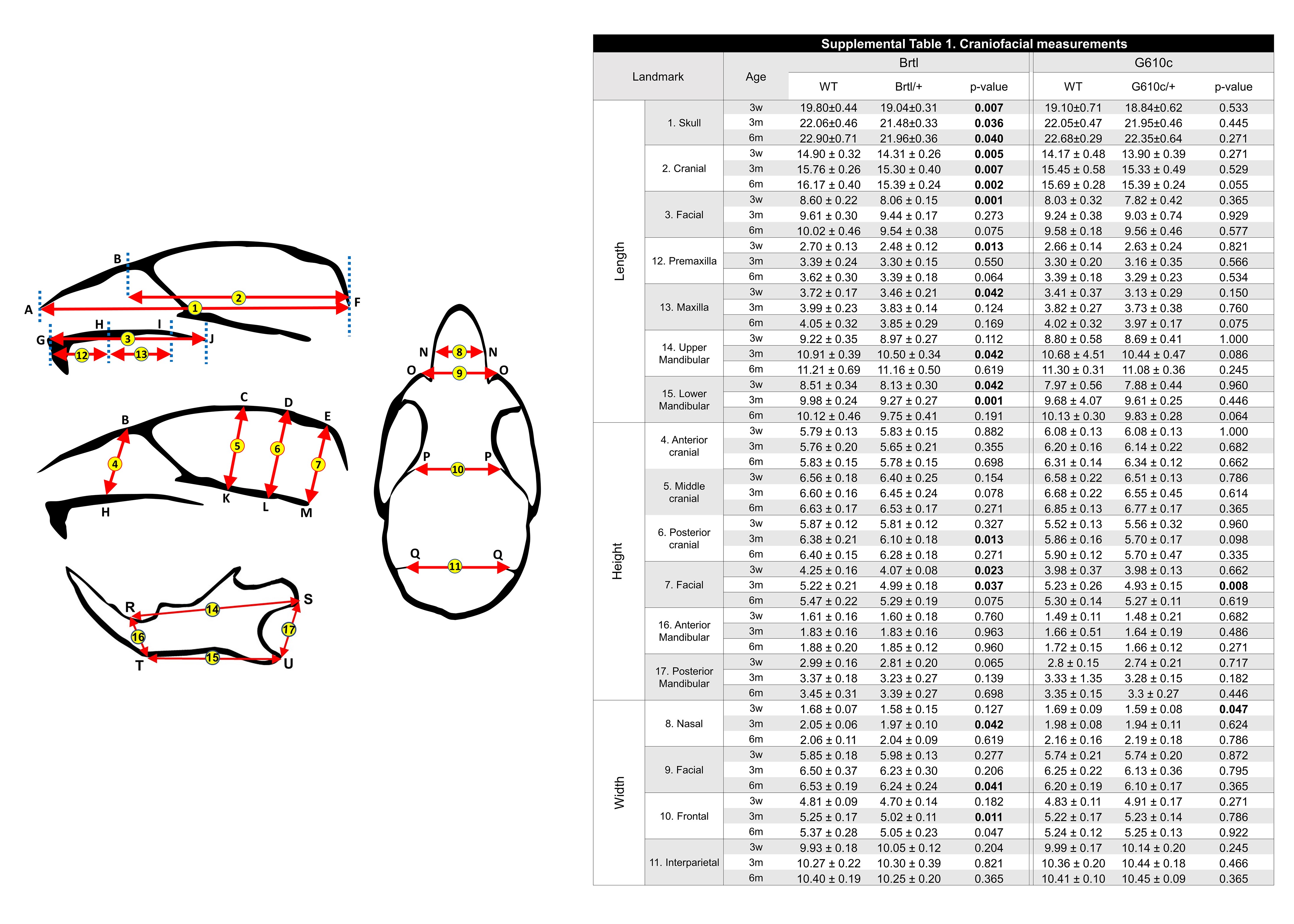

Supplement: Figure_S3_ziad004 [file figure_s3_ziad004.jpeg]

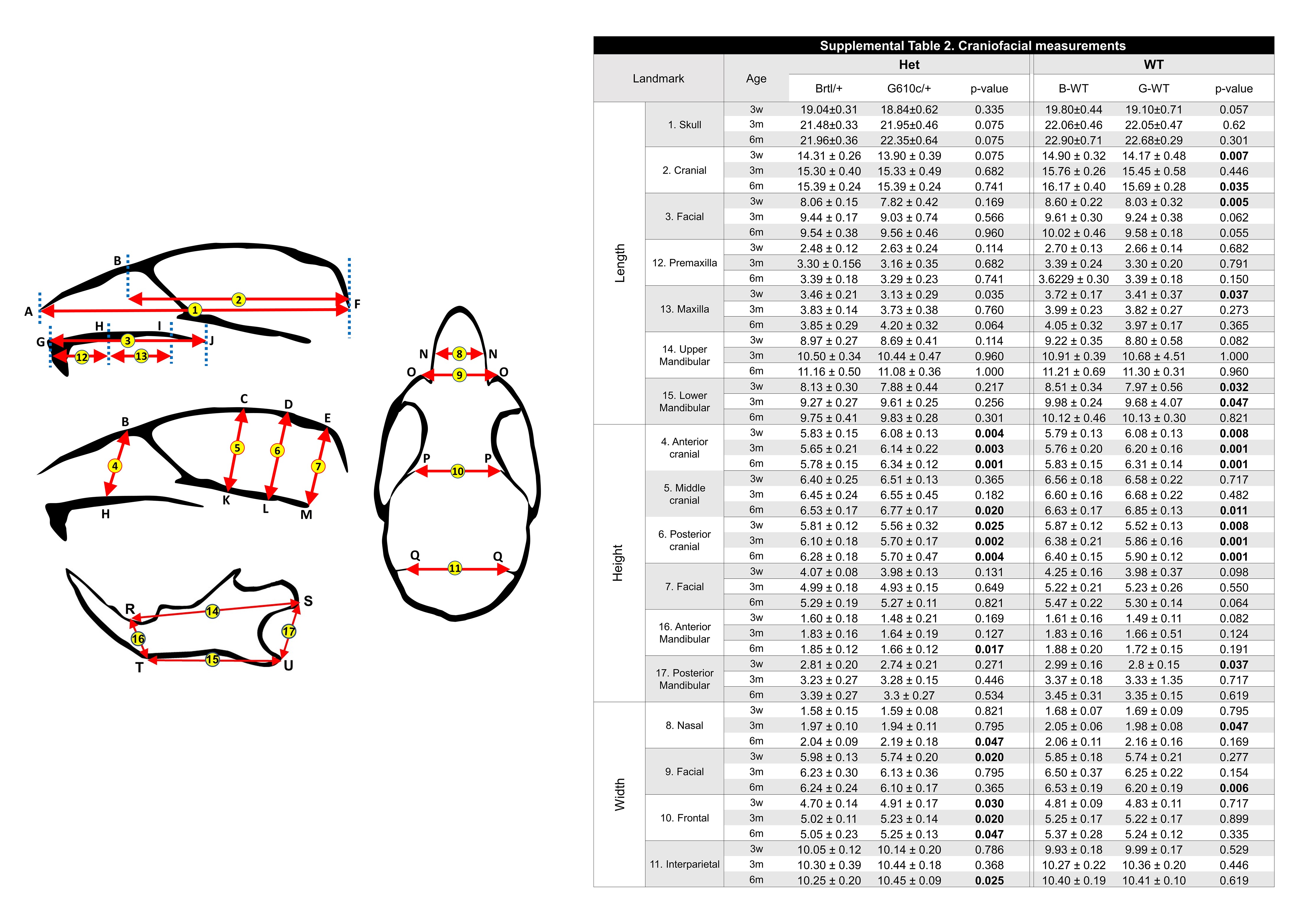

Supplement: Figure_S4_ziad004 [file figure_s4_ziad004.jpeg]

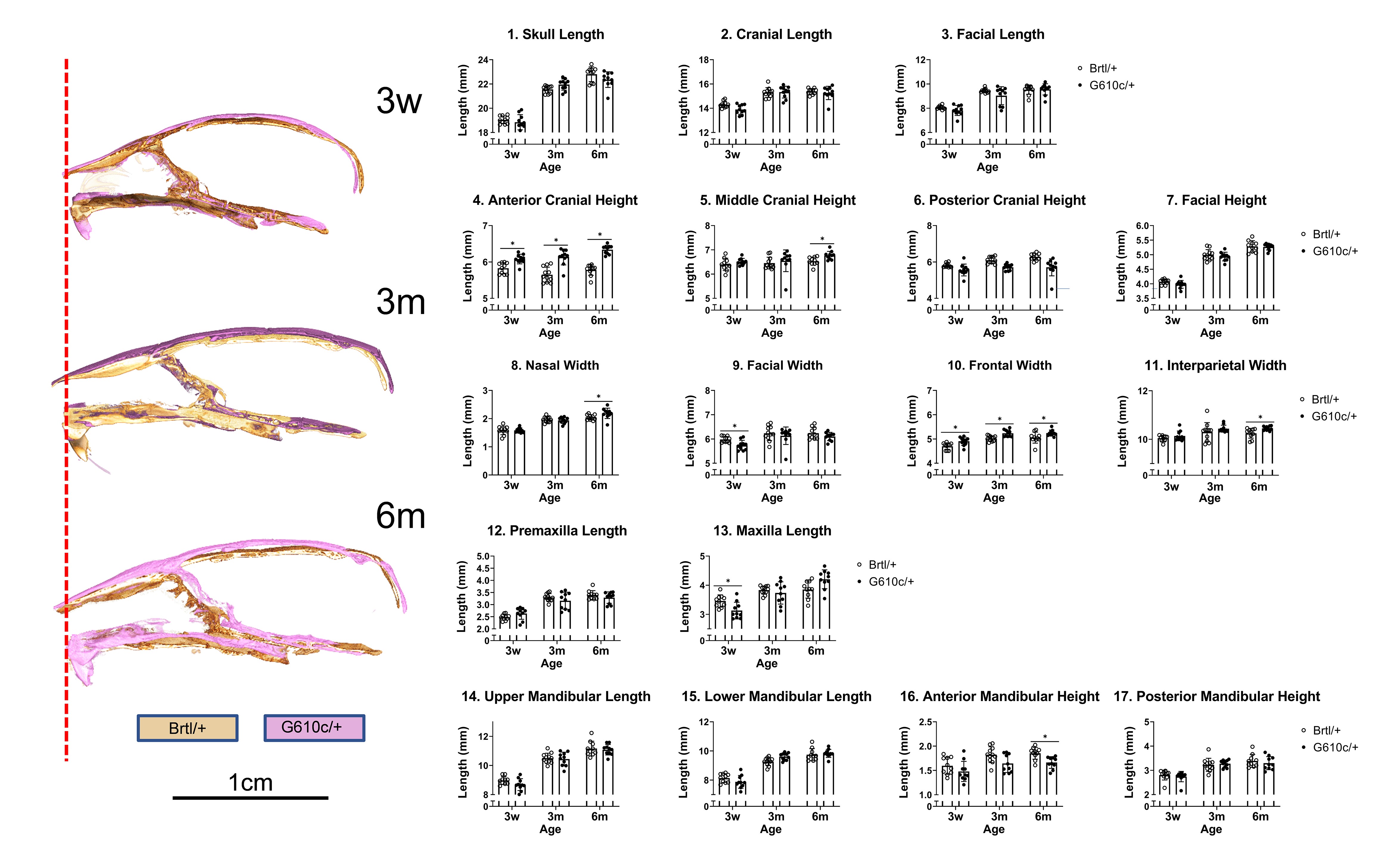

Supplement: Figure_S5_ziad004 [file figure_s5_ziad004.jpeg]

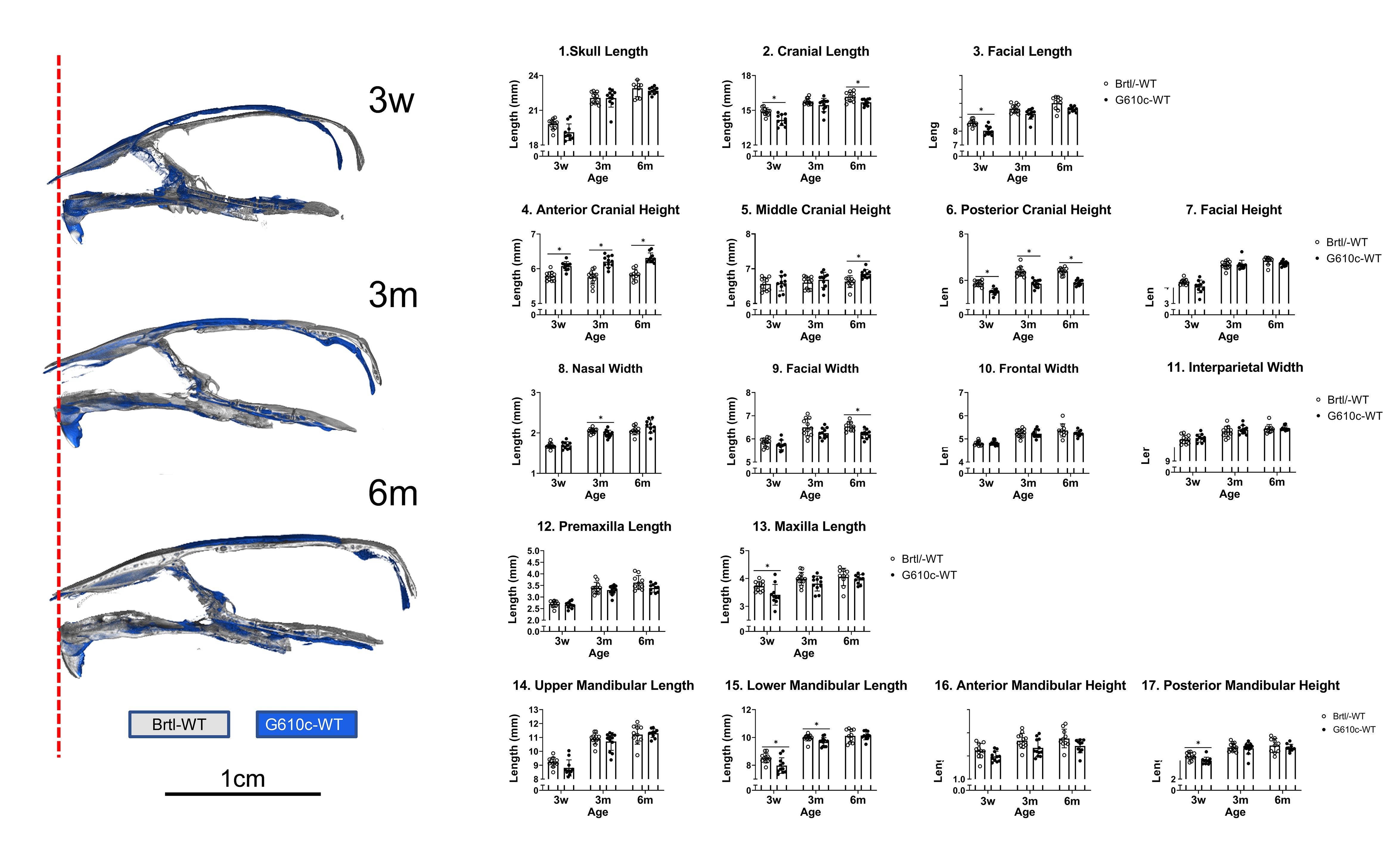

Supplement: Figure_S6_ziad004 [file figure_s6_ziad004.jpeg]

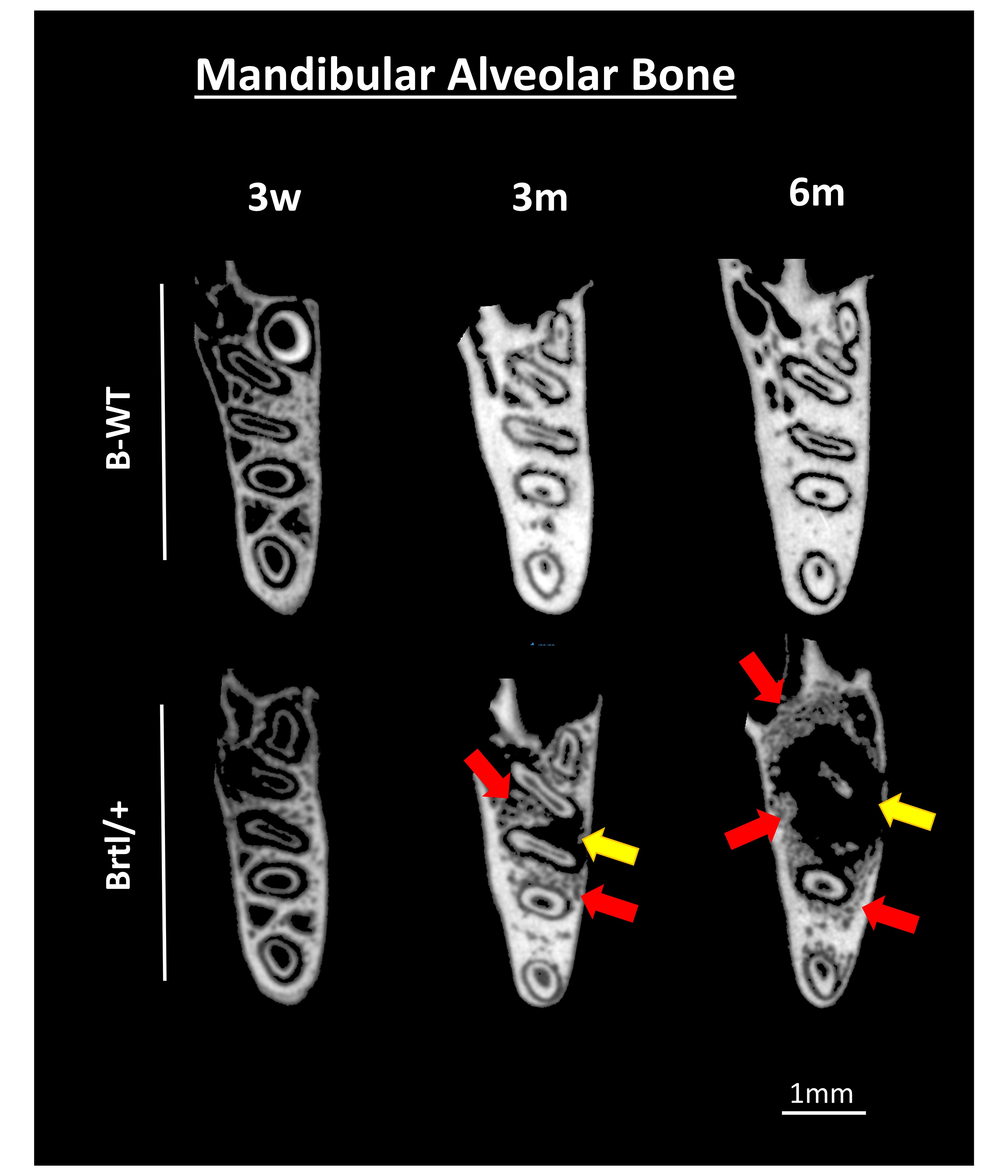

Supplement: Figure_S7_ziad004 [file figure_s7_ziad004.jpeg]

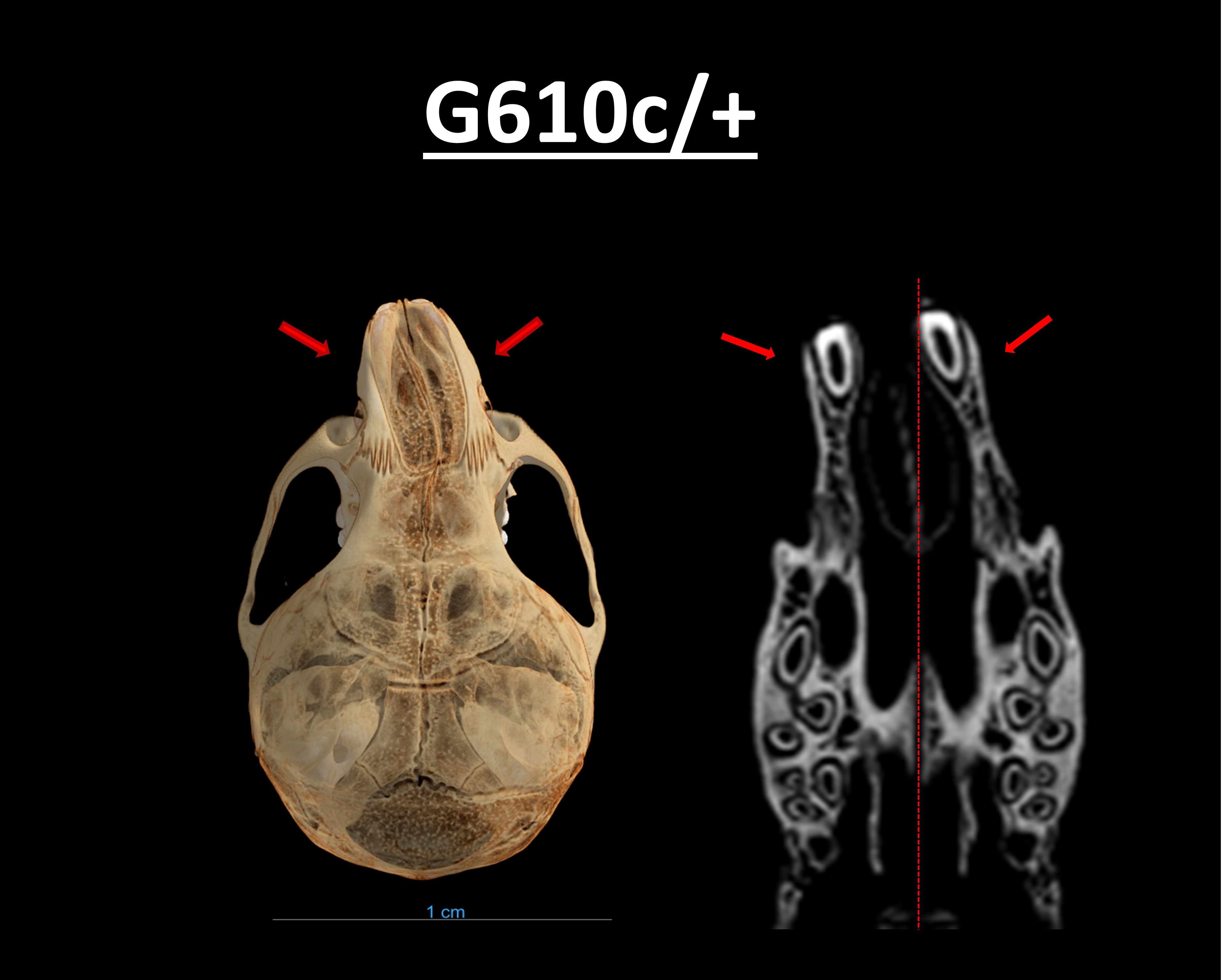

Supplement: Figure_S8_ziad004 [file figure_s8_ziad004.jpeg]
